# Explainable Radiomics-Based Model for Automatic Image Quality Assessment in Breast Cancer DCE MRI Data

**DOI:** 10.3390/jimaging11110417

**Published:** 2025-11-19

**Authors:** Georgios S. Ioannidis, Katerina Nikiforaki, Aikaterini Dovrou, Vassilis Kilintzis, Grigorios Kalliatakis, Oliver Diaz, Karim Lekadir, Kostas Marias

**Affiliations:** 1 Computational BioMedicine Laboratory (CBML), Foundation for Research and Technology—Hellas (FORTH), 70013 Heraklion, Greecedovrou@ics.forth.gr (A.D.);; 2 Department of Biomedical Sciences, Radiology-Radiotherapy Sector, University of West Attica, 12243 Athens, Greece; 3 Medical School, University of Crete, 71003 Heraklion, Greece; 4 Artificial Intelligence in Medicine Lab (BCN-AIM), Department of Mathematics and Computer Science, Universitat de Barcelona, 08007 Barcelona, Spain; oliver.diaz@ub.edu (O.D.);; 5 Institució Catalana de Recerca i Estudis Avançats (ICREA), Passeig Lluís Companys 23, 08010 Barcelona, Spain; 6 Department of Electrical and Computer Engineering, Hellenic Mediterranean University, 71410 Heraklion, Greece

**Keywords:** image quality assessment, DCE MRI, radiomics, breast imaging, objective quality metrics, machine learning, explainability

## Abstract

This study aims to develop an explainable radiomics-based model for the automatic assessment of image quality in breast cancer Dynamic Contrast-Enhanced Magnetic Resonance Imaging (DCE-MRI) data. A cohort of 280 images obtained from a public database was annotated by two clinical experts, resulting in 110 high-quality and 110 low-quality images. The proposed methodology involved the extraction of 819 radiomic features and 2 No-Reference image quality metrics per patient, using both the whole image and the background as regions of interest. Feature extraction was performed under two scenarios: (i) from a sample of 12 slices per patient, and (ii) from the middle slice of each patient. Following model training, a range of machine learning classifiers were applied with explainability assessed through SHapley Additive Explanations (SHAP). The best performance was achieved in the second scenario, where combining features from the whole image and background with a support vector machine classifier yielded sensitivity, specificity, accuracy, and AUC values of 85.51%, 80.01%, 82.76%, and 89.37%, respectively. This proposed model demonstrates potential for integration into clinical practice and may also serve as a valuable resource for large-scale repositories and subgroup analyses aimed at ensuring fairness and explainability.

## 1. Introduction

Assessing image quality is a task of utmost importance both for conventional diagnosis and for Artificial Intelligence (AI)-assisted decision support systems. Efforts have been made to assess the effect of image quality on performance metrics [1]. Moreover, ongoing efforts to build large image repositories show great interest in automatically identifying the level of data quality, in all contributed datasets, to be aligned with the principles and optimal practices of AI in medicine [2]. Acquiring information on image quality is also closely related to traceability as a measure to describe model input and relate it to the output. Robustness is also examined with respect to image quality fluctuations which are expected and well received within large data volumes but, on the other hand, should be registered in a consistent manner. To this end, evaluation of image quality plays a major role in the recommended frame of trustworthy AI use in medicine, as defined within the FUTURE AI framework [3,4], in order to improve adaptness and deliver impactful service to the patients.

In a conventional human expert-based diagnostic environment, assessment on image quality happens almost intuitively by the expert who will either reject the diagnosis based on an image of unaccepted quality or will ignore non-significant image degradation factors. However, the development of large imaging infrastructures requiring massive data contributions intensify the need for automated tools that are able to recognize severely degraded images which may compromise the general ability of the dataset to deliver accurate and trustworthy results. On the other hand, subtle degradations and artifacts are expected and, to large degree, well received as part of a real-world dataset, characterized by heterogeneity, which is also an aspect of AI model generalizability. Subtle degradation refers to images presenting sub-optimal characteristics, as opposed to severe degradation which potentially jeopardizes diagnosis.

For this reason, the task of image quality assessment can be decomposed on the important pillars below. Firstly, to assess image quality and blacklist images of unacceptable quality, and secondly to identify and rank images of adequate quality.

Breast Dynamic Contrast-Enhanced Magnetic Resonance Imaging (DCE-MRI) sequence constitutes the cornerstone of breast diagnosis at high-risk population and also for disease progression, as the type of enhancement is a basic criterion and as such, certain quality criteria are recommended by breast imaging societies. DCE-MRI is a technically challenging sequence, especially in the anatomic region of the breast for a number of reasons. Firstly, the solid frame of the coil with the variable breast size allows for different types of mispositioning the human body in the coil, being either far from the receiver coil in the case of small breastσ causing low Signal-to-Noise Ratio (SNR) or touching the coil in the opposite case causing incomplete inhomogeneous fat signal suppression and thus low Contrast-to-Noise Ratio (CNR). Moreover, the need for keeping temporal requirements of the dynamic acquisition does not allow for optimal spatial resolution settings or if spatial resolution is uncompromised, noisy images may occur. The nature of DCE-MRI gradient echo acquisitions also favors a number of artifacts, such as zebra artifacts, although usually presented not within the useful field of view.

Other challenges related to breast MRI and not to any other anatomy is the significant tissue–air interface area enhancing susceptibility related degradation, the massive heart flow artifact present in the field of view, and also the presence of anatomic areas in the image not covered by receiver coil, such as the spine, presented inevitably with high noise over signal. These unique characteristics render breast DCE-MRI a very challenging instance of breast MRI acquisition.

### Related IQA Works

In the literature, works assessing Internal Quality Assurance (IQA) in MRI with AI techniques are mainly focused on deep learning techniques in different parts of the human body that lack explainability due to the nature of deep learning itself. More specifically, Piccini et al. developed a deep convolutional neural network (IQ-DCNN) to automatically assess the quality of 3D whole-heart MRI data, mimicking expert human evaluations [5]. IQ-DCNN showed strong agreement with human assessments and effectively tracked image quality improvements during reconstruction. In addition, Lin et al. developed a deep learning algorithm to assess prostate MRI quality and found that higher-quality T2-weighted images significantly improved the specificity of detecting extraprostatic extension (EPE) in prostate cancer [6]. Also, they found that high-quality T2W images were associated with more accurate prediction of EPE at final pathology. Furthermore, Stepien et al. proposed a novel no-reference deep learning method for MR IQA by fusing complementary network architectures and leveraging multi-level features using two image quality benchmark datasets with various body locations such as spine, knee, shoulder, brain, wrist, hip, pelvis, elbow, and ankle [7]. This deep learning method outperformed existing techniques, showing strong correlation (Pearson’s r = 0.9062) with radiologists’ quality ratings.

A review paper of 2025 by Herath et al. regarding IQA in MRI highlighted two deep learning methods for the detection of motion artifacts in cardiac and fetal brain MRI addressed by multi-task learning with k-space motion artifact augmentation and semi-supervised models, respectively [1]. The review also indicated an IQ-DCNN, for whole-heart MRI reconstruction monitoring and an Optimized Deep Knowledge-based No-reference Image Quality Index (ODK-NIQI) for denoising.

Works in the literature concerning machine learning methodologies are limited. For example, Chabert et al. developed a machine learning method to classify MR image quality based on radiologist perception, focusing on lumbar MR data [8]. In particular, the authors used the Support Vector Machine (SVM) classifier with features extracted from 95 exams and achieved an average recall of 82% and AUC of 77%, showing promising results for future real-time IQA. There is also a work of Ma et al. that incorporated traditional image quality metrics such as Peak Signal-to-Noise Ratio (PSNR) and Structural Similarity Index (SSIM), Blind/Referenceless Image Spatial Quality Evaluator (BRISQUE), and Natural Image Quality Evaluator (NIQE) and correlated them with the opinion of thirteen expert radiologists [9]. The findings highlight the importance of aligning objective IQA models with radiologist judgments to enhance clinical relevance in MRI quality assessment. However, the aforementioned study relied on simulated artifacts and is constrained by the unique challenges of medical image data used in clinical practice.

Most recently, efforts have been put on brain MRI [10,11,12], while few are anatomy agnostic allowing for the assessment of breast MRI acquisitions [13]. To the author’s knowledge this work is the first effort to develop a breast DCE-MRI specific explainable model for automatically assessing image quality, embracing the specific requirements of breast anatomy and DCE-MRI acquisition characteristics.

## 2. Materials and Methods

### 2.1. Patient Population/Imaging Protocol

For the needs of this study the MAMMA-MIA dataset [14] was used in order to classify images of high or low quality. The dataset comprised 4 public datasets (Duke, ISPY1, ISPY2, Nact) resulting in 1506 homogenized DCE-MRI images with variable protocols. Further information about patient characteristics and imaging protocols can be found in Table 1 and Table 2 [14].

### 2.2. Image Quality Labeling

**Figure 1:**
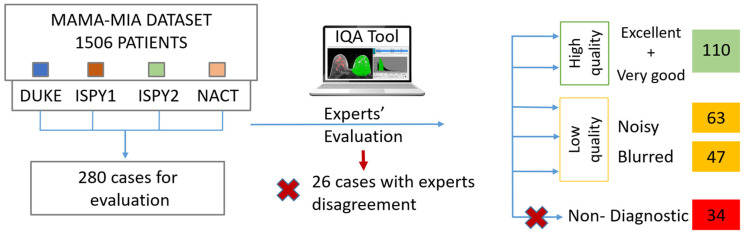
Image quality labeling process for model training. The red X indicates the cases that were excluded.

**Figure 2:**
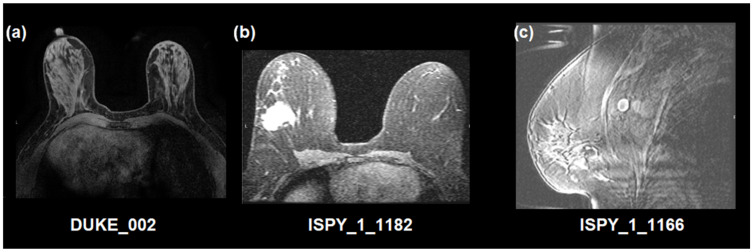
Image quality labeling examples. (**a**) High-quality image, (**b**) low-quality image (noisy), (**c**) low-quality image of unacceptable quality (non-diagnostic, excluded from the analysis, blurred, also presenting a number of artifacts, i.e., zebra, aliasing, and susceptibility). Patients’ identifiers are presented at the bottom of the image.

With the support of an in-house built IQA tool [15], two experts (a radiologist and a medical physicist with 11 and 13 years of experience, respectively) annotated the DCE-MRI series with respect to their quality. Randomly selected images at equal volumes from each one of the four datasets were included in the dataset for further analysis (280 cases). We considered a minimum threshold of 110 patients to populate each one of the classes, also achieving consensus among the two experts with respect to their decision. The high-quality class (labeled 0) contained excellent or very good images, as assessed in a series base, while the latter contained noisy, blurry, low contrast, or images presented with severe artifacts grouped, indifferently of the cause to form a single, low-quality class (labeled 1). Furthermore, 26 cases with expert disagreement, as well as 34 cases described as non-diagnostic by at least one expert, were excluded. The overall image quality labeling process is depicted in Figure 1. In addition, as an example, a visual representation of image quality of the studied dataset is presented in Figure 2.

### 2.3. Experimental Set Up

**Figure 3:**
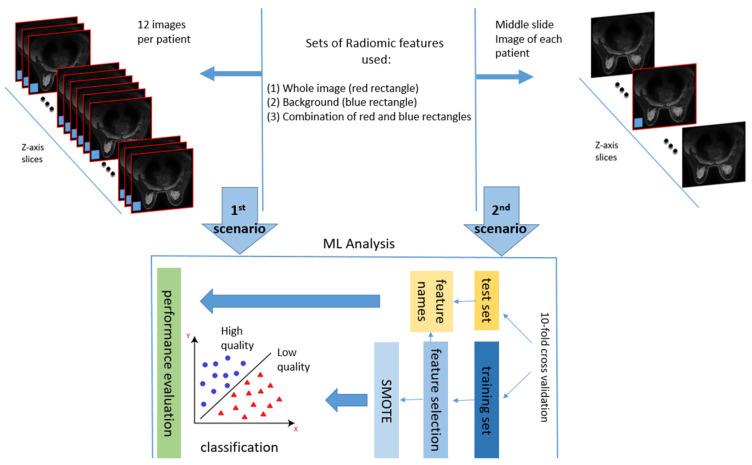
Experimental set up—workflow of IQA showing the two scenarios explored.

In this work, image quality was assessed on a patient level basis in two different scenarios (Figure 3). Firstly, image quality was examined in 12 slices of the first post contrast image of the DCE-MRI sequence for every patient. Specifically, 6 equidistant images from the middle third of the data acquisition volume and 3 from the top and bottom third of the volume, respectively. The rationale behind the selection of a subset of images across the series was to address the possibility of having local degradations in a sub-region of anatomy, related to coil sensitivity variation, patient anatomy, or presence of foreign objects in the body such as surgical clips. Secondly, image quality was examined only at the middle slice in the z-axis of the first post-contrast image to reduce computational effort. The middle slice was chosen as strongly representative of a global notion of image quality within a series acquired by a certain imaging protocol set up.

For both scenarios two regions of interest (ROI) were examined, i.e., the whole image (red rectangle in Figure 3) and the background region (blue rectangle in Figure 3), which were used for feature extraction as separate regions as well as combined in a single set of features. It is noteworthy to mention that the background ROI was obtained automatically in a square region (⅛ of image width and ⅛ of image height) pixels in each of the four corners of the image. Since the protocols consisted of both axial and sagittal images, the noise region was determined based on the lowest SNR among the two squares and the constraint not to be diagonal. This criterion was able to locate two background regions per slice irrespectively of the acquisition plane orientation.

### 2.4. Radiomics Extraction

The first post-contrast images were standardized using z-score normalization based on the whole image to adjust for the arbitrary intensity values of the MRI scans. The radiomics analysis was initiated by extracting 819 radiomic features from the whole image as well as the defined background region. Moreover, two objective No-Reference (NR) image quality metrics (BRISQUE and total variation) were calculated from PyTorch Image Quality (PIQ) library version 0.8.0 [16] to complement the analysis, adding up to a final set of 821 features. The radiomic features were extracted using the open-source python library PyRadiomics version 3.0.1 [17], in compliance with the Image Biomarker Standardization Initiative (IBSI) guidelines. Features were extracted from the original images as well as from images processed with the wavelet filter and the Laplacian of Gaussian (LoG) filter, applying 4 different values of sigma (2, 3, 4, and 5) to highlight various image textures. A total of 819 radiomics features were extracted for each image–segmentation pair, including first-order features (*n* = 162) and higher-order textural features (*n* = 657). The textural features consisted of Gray Level Co-occurence Matrix (GLCM) features (*n* = 198), Gray Level Dependence Matrix (GLDM) features (*n* = 126), Gray Level Run Length Matrix (GLRLM) features (*n* = 144), Gray Level Size Zone Matrix (GLSZM) features (*n* = 144), and Neighboring Gray Tone Difference Matrix (NGTDM) features (*n* = 45). Lastly, the abovementioned radiomic features were calculated after applying the Fixed Bin Size (FBS) discretization technique, recommended by the IBSI with two different bin widths (1 and 54) in order to see the impact of image intensity grouping in the quality classification and computational time. The bin width of 54 was determined by calculating the mean intensity range within the ROI and dividing by a Fixed Bin Number (FBN) of 64, a commonly accepted value [18,19].

#### 2.4.1. Classification—Machine Learning Pipeline

To identify which features are the most dominant to assess image quality, a radiomics/machine learning pipeline was developed using a variety of widely used classifiers, including Logistic Regression, Support vector Machine (SVM) using the radial basis function kernel, K-Neighbors Classifier (KNN), Random Forest Classifier, AdaBoost Classifier, and GaussianNB. All classifiers were trained and evaluated in a 10-fold cross-validation scheme on the extracted features. Data stratification was applied on a patient basis across folds, avoiding sample selection bias and overfitted models.

To evaluate and compare the performance of image quality classification, a variety of common evaluation metrics were deployed. More specifically, for every fold, sensitivity, specificity, accuracy (ACC), and the area under the curve (AUC) along with their standard deviations (SD) were calculated on the unseen testing sets in each fold. Furthermore, for the first scenario, meaningful for patient-level evaluation, predicted probabilities from all 12 slices belonging to the same patient were aggregated using a soft voting scheme (i.e., averaging probabilities across slices/samples). Final class predictions were obtained by thresholding the averaged probability at 0.5.

The feature selection process relied on a three-step scheme. Firstly, a variance threshold = 0.05 was applied to remove features with zero variance (constant features). Secondly, ANOVA (analysis of variance) was used as a univariate method to reduce noisy information in the feature space. Thirdly, logistic regression based on the L1 penalty was used by evaluating the magnitude and significance of coefficients to force less important feature coefficients to zero (LogisticRegression with inverse of regularization strength C = 0.5). More information about the feature dimensionality reduction can be found in [20,21].

#### 2.4.2. Explainability Analysis

To evaluate the interpretability of the image quality models, Shapley Additive Explanations (SHAP) were employed to provide insights into individual predictions. SHAP values are model-agnostic, making them suitable for explaining any type of machine learning algorithm. Rooted in game theory, SHAP assigns an importance score to each feature based on its contribution to the overall prediction, similar to how each player’s role is assessed in a cooperative game. In the context of machine learning, these values quantify the influence each feature has on the model’s output. The Shapley value represents the average contribution of a given feature across all possible feature combinations [22,23].

Feature-level interpretability is crucial, especially for uncovering hidden relationships in complex data that are not immediately apparent. In this study, summary plots were generated based on the fold with the highest performance in terms of ACC and AUC for all image quality scenarios.

## 3. Results

A number of classifiers were deployed to accomplish the classification of an image based on the calculated image features into low- and high-quality classes. For both of the aforementioned scenarios, i.e., training the model either with a set of 12 images per patient across the series or with a single image in the center of the imaging volume for each patient, the performance metrics, using the whole image, are presented in Table 1.

Results in Table 1 were calculated at a patient level. For all cases of 12-slice evaluation a probability-based voting was utilized, i.e., the mean probability for each patient was calculated as the average probability across all 12 slices according to a soft voting approach, i.e., a threshold of 0.5 probability for each class (high/low quality) assigned the high-quality/low-quality label to the whole set of 12 slices of this individual patient. This action also alleviated the imbalance in the number of examined slices for each patient case, as in the first scenario the prediction would be ambiguous for a significant degree of discordance among slices. Moreover, if only cases with all slices converged to the same decision were used, the number of instances participating in the performance evaluation would be above one order of magnitude larger in the 12-slice evaluation option. In addition, the results for the second scenario (features used only from the middle slice) are presented in Table 2.

From the results presented in Table 1 and Table 2, it is evident that single slice analysis has superior performance than the 12 slices analysis based on the best performing classifier (Random Forest), apart from the apparent reduction in computational cost. Also, the SVM converges with the same result, i.e., the single slice analysis outperforms the 12-slice analysis.

**Figure 4:**
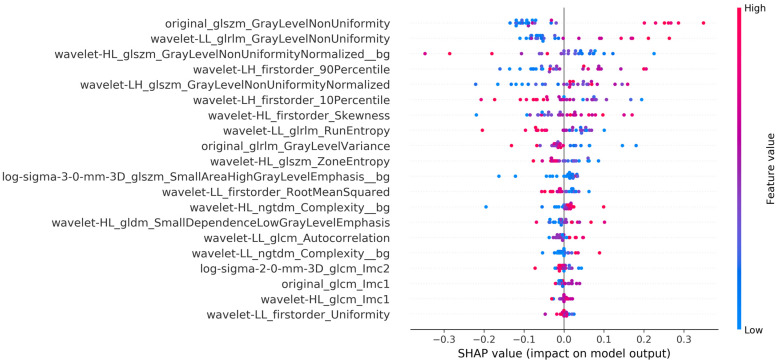
Best model’s (middle slice and combination of radiomics from whole image and background) summary plot of the SHAP analysis explaining the impact/importance of each feature to classify between high and low image quality with the SVM classifier. It has to be noted that feature names that end with ‘bg’ refer to the background ROI features.

As a next step, the explainability analysis for the single slice in the middle of the acquisition volume was performed, utilizing the combined radiomics from the whole image and the background. Thus, the summary plot of SHAP values for the best model is presented in Figure 4.

## 4. Discussion

This work focused on the development of a free-of-anatomy breast DCE-MRI specific tool for automatic detection of image quality. The superiority of the single slice model over the 12-slice model (scenario 1) that runs across the whole imaging volume can be attributed to the fact that the middle slice is dominantly occupied by patients’ anatomy with radiological/diagnostic interest, while the rest of the anatomy that is undercovered by imaging coils remains the same. Moreover, for certain patients’ anatomic characteristics, i.e., small breast volume, the upper and lower part of the acquisition volume were not indicative of image quality and possibly contaminated the analysis with non-relevant information.

**Figure 5:**
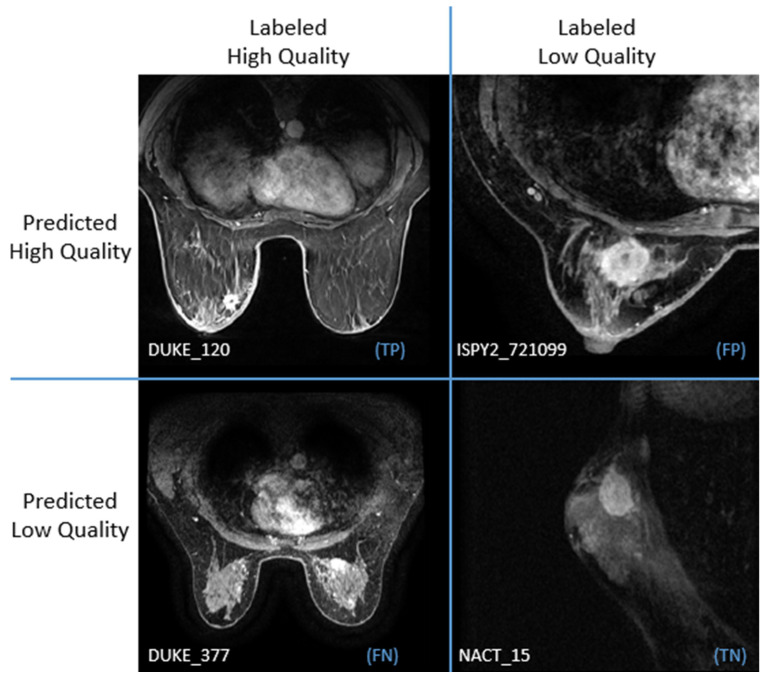
Visual confusion matrix for predicted and labeled image quality. Patients’ identifiers are presented at the bottom of the image. Annotations in parentheses with blue color TP/FP/FN/TN denote: True positive/False Positive/False Negative/True Negative.

By using the SVM classifier and the whole image and background features (scenario 2) the model achieved performance metrics (ACC/AUC) of (82.76 ± 6.28/89.37 ± 4.64), respectively, concluding to a percentage of 20% of misclassifications in the testing set (i.e., 8% of images of high quality according to the expert classified as low by the model, and 12% of the images evaluated as low quality by the expert but where classified as high quality by the model). On the opposite side, the agreement between the expert and the model was achieved in 80% of the cases. Figure 5 shows images of agreement and disagreement between the expert and the model in a visual confusion matrix comparing expert and model evaluation.

From the metrics presented in Table 1 and Table 2 it is obvious that the background region has inferior performance than the whole image, which can be attributed to the fact that the anatomy of interest is excluded from the examined region. However, given the fact that it focuses on a part of the image that isolates and thus is able to capture noise levels and presence of artifacts (most prominent in anatomy free regions), it was considered meaningful to adapt the final outcome and produce a more complex model that uses both the whole image region and the noise region synergistically. To achieve this, we produced a model by using the radiomic features from the whole image along with those from the background region. The final model had double the number of features compared to the ones used before.

The explainability results (Figure 4) show that gray level Non-Uniformity, both in the Wavelet decomposed as well as in the original image, are among the top three features, which is aligned with what is intuitively expected to distinguish a blur indicating inhomogeneity across intensity values. The participation of the background region confirms the initial assumption that the air region should be included and can improve the overall performance.

Also, zone entropy metrics play an important role for the whole image which is also a metric of uncertainty or randomness, which can also be associated with characteristics of heterogeneous texture patterns. For the background regions the metrics showing a powerful effect on the model decision is the NGTDM Complexity, which quantifies differences between a pixel’s gray value and the mean gray value of its neighbors, showing uniformity in anatomy free regions which can probably capture sensitivity uniformities or presence of artifacts.

It is of importance that the objective NR metrics did not appear in the SHAP analysis to have a strong effect on the decision. For this outcome, the non-medical orientation of NR metrics might be an explanation since the images of the breast region are inherently expected to have large areas of blurring and low SNR (massive heart flow artifact, back and spine region without coil coverage, absent anatomy in many slices for some body types or pathologies) and thus the DCE breast MRI image has a very precise definition of quality which is different from natural images. Moreover, DCE-MRI is a dynamic fast acquisition which is error-prone by design in order to achieve the desired temporal resolution to capture contrast agent dynamics. It is expected, therefore, that even excellent images have areas of sub-optimal quality due to the nature of the acquisition and thus the NR non-medical metrics are not effective, as also observed by Kastryulin et al. [24]

The experimentation with bin size was initiated by the thought that pixel intensity grouping might obscure heterogeneities in pixels varying at a small degree and that could have a negative effect if those pixels are neighboring, i.e., flattening the contrast. However, when omitting this grouping of signal intensities, i.e., using bin width = 1 (no discretization), the results were not improved. Hence, the discretization process was applied using the FBS technique, as recommended by the IBSI, with a bin width of 54. This value was chosen based on the signal intensities in order not to raise the computational cost without a measurable benefit.

The decision to use a more complex model with the whole image radiomics and background radiomics instead of the best performing model of the whole image was based on the rationale that in MR images the background region is the one most indicative of noise levels and also is the region accommodating most of the artifacts or highlighting them as they are more prominent in a low signal region. The whole image analysis is indispensable as blurring can only be seen in the imaging volume. Based on this rationale, a complex model using both areas was preferred, since the computational cost was not considered prohibitive.

Based on the presented results and the abovementioned criteria, we concluded the final settings of the proposed model of this work. The tool achieved similar sensitivity and specificity values, meaning that possible misclassifications are equally possible either to have false positives (bad images in the high-quality class) or false negatives (good images in the low-quality class). Since these events are sporadic, given the achieved performance, the tool delivers a trustworthy output for statistical descriptive features given a large dataset. This, in turn, is a valid metric of dataset value in a multi-centric and/or multi-vendor diverse protocol setting. In a per-patient classification application, the tool is able to capture consistent factors of image degradation, such as a coil failure, a room temperature modification, or any other factor affecting the anatomy representations. This can serve as an alert mechanism and be an effective tool for service auditing in clinical sites.

The feature selection method substantially reduced the original 819 features to approximately 15–20 highly informative features used for classification. Such dimensionality reduction not only mitigates overfitting risks associated with small sample sizes (110 per class) but also enhances the model’s generalization and computational efficiency. Therefore, more aggressive reduction was unnecessary, as the resulting feature subset already demonstrated strong discriminative performance and stability across folds.

The feature extraction process required approximately 2500 milliseconds (2.5 s) per slice when executed on a Linux server equipped with dual Intel(R) Xeon(R) Gold 5220R CPUs operating at 2.20 GHz and 1 TB of RAM. The subsequent classification phase required approximately 1–2 s per slice, depending on the specific cross-validation fold. This approach demonstrates potential for real-time clinical implementation, as the feature selection stage reduces the feature set to only 15–20 key descriptors. Consequently, the evaluation of new cases in terms of image quality can be performed efficiently, with predictions based on a compact and highly informative subset of features, ensuring both rapid and accurate classification.

To extend the best of our models into a real-world clinical scenario, the model combining the whole image and background radiomics was also tested on the whole MAMA-MIA dataset to predict each image as high or low quality. To be noted the MAMA-MIA dataset comprises four publicly available datasets named DUKE ISPY_1, ISPY_2, and NACT. Interestingly, for each of these subsets of MAMA-MIA’s dataset we found the percentage of low-quality images for NACT 7/63 (11%), ISPY1 26/171 (15%), DUKE 74/283 (26%), and ISPY2 97/972 (10%). The measured percentage of low-quality images and the heterogeneity among different protocols are within the ranges expected from clinical experience that the evaluators of this work (radiologist and medical physicist) encounter in clinical practice and evaluate as acceptable within a realistic working environment.

Since the image quality per se might not be the question of utmost clinical importance, as a future step the image quality classification task can be correlated with tangible benefits for the patient, as the final and most important recipient of any medical action. Such a final outcome can stem from achieving better performance in a treatment prediction model or a risk estimation outcome. Moreover, the dataset deployed during this work was limited in volume. In the case of a larger examined dataset for expert labeling and training it could enable a more detailed classification of images with respect to the etiology of degradation, i.e., specifying the dominant factor of quality compromisation. Another limitation is the recruitment of only two experts of different expertise. The analysis would have improved if more than two experts assessed the quality to also address interobserver disagreements more efficiently. It also has to be noted that the deployment of such a tool in other anatomies or modalities is not constrained by the general framework of this work and thus can be configured to have anatomy or modality specific modules.

## 5. Conclusions

To summarize, an automatic detection of image quality assessment model was specified for first pass DCE-MRI data. The model was optimized for performance and computational cost. This model can be integrated into real clinical practice but can also serve as a valuable tool for large volume repositories and subgroup analysis for fairness and explainability control. As image quality per se is not a clinical endpoint, it can also be deployed for the benefit of the end user, i.e., the patient to correlate image quality with treatment response prognosis or disease classification performance metrics.

## Figures and Tables

**Table 1:** Performance evaluation metrics for classification of images as high- or low-quality images for a number of different classifiers. The results presented are for the whole image, background region, as well as the combined analysis with features from both areas. Values correspond to mean ± standard deviation (SD) and are evaluated on the 12-slice per patient option (1st scenario).

Set of Features Used	Classifier	Sensitivity (±SD)	Specificity (±SD)	ACC (±SD)	AUC (±SD)
Whole Image	Logistic Regression	69.99 ± 10.76	78.91 ± 7.32	74.54 ± 4.14	83.49 ± 5.07
	SVM	75.34 ± 14.65	80.77 ± 11.01	78.11 ± 7.27	86.15 ± 4.04
	KNN	66.21 ± 12.78	81.80 ± 8.04	74.06 ± 7.01	82.26 ± 5.40
	Random Forest	74.38 ± 11.37	83.59 ± 10.05	79.03 ± 5.85	85.96 ± 4.68
	AdaBoost	73.42 ± 14.08	78.16 ± 10.74	75.91 ± 5.66	83.46 ± 5.20
	Gaussian NB	60.78 ± 14.21	67.10 ± 16.76	64.10 ± 4.80	72.79 ± 6.41
Background	Logistic Regression	66.67 ± 12.43	87.23 ± 7.86	80.42 ± 5.24	85.35 ± 4.24
	SVM	69.94 ± 15.38	80.70 ± 7.33	77.29 ± 7.47	80.82 ± 8.16
	KNN	66.67 ± 12.43	74.24 ± 11.99	71.88 ± 9.00	78.64 ± 9.87
	Random Forest	52.98 ± 17.40	90.80 ± 3.27	78.48 ± 6.66	85.25 ± 7.16
	AdaBoost	59.23 ± 23.61	86.20 ± 9.45	77.35 ± 8.42	80.03 ± 10.23
	Gaussian NB	31.25 ± 19.25	94.37 ± 6.18	73.66 ± 8.33	60.89 ± 19.24
Whole Image + Background	Logistic Regression	71.15 ± 7.45	79.95 ± 11.06	75.62 ± 6.03	83.73 ± 5.66
	SVM	70.19 ± 8.10	81.59 ± 8.14	76.07 ± 6.15	83.13 ± 6.80
	KNN	61.54 ± 10.88	86.26 ± 4.26	74.18 ± 5.08	78.96 ± 6.51
	**Random Forest**	**69.23 ± 9.42**	**81.66 ± 11.38**	**75.59 ± 9.01**	**87.30 ± 7.74**
	AdaBoost	73.08 ± 12.76	81.66 ± 10.81	77.53 ± 9.82	84.59 ± 7.20
	Gaussian NB	67.31 ± 12.01	49.45 ± 15.18	58.17 ± 10.54	67.34 ± 9.84

Bold row indicates the best performance.

**Table 2:** Performance evaluation metrics for classification of images as high- or low-quality images for a number of different classifiers. The results presented are for the whole image, background region, as well as the combined analysis with features from both areas. Values correspond to mean ± standard deviation (SD) and are evaluated on the single (middle) slice per patient option (2nd scenario).

Set of Features Used	Classifier	Sensitivity (±SD)	Specificity (±SD)	ACC (±SD)	AUC (±SD)
Whole Image	Logistic Regression	72.32 ± 9.06	74.11 ± 13.80	73.21 ± 7.99	80.04 ± 6.97
	SVM	67.86 ± 11.29	78.57 ± 13.36	73.21 ± 9.62	84.63 ± 5.11
	KNN	39.29 ± 17.86	91.07 ± 11.15	65.18 ± 6.12	78.16 ± 5.50
	Random Forest	77.68 ± 6.62	77.68 ± 16.15	77.68 ± 6.86	84.41 ± 6.00
	AdaBoost	70.54 ± 13.09	75.89 ± 11.26	73.21 ± 3.99	81.51 ± 4.98
	Gaussian NB	54.46 ± 17.83	76.79 ± 10.56	65.63 ± 7.97	75.77 ± 7.12
Background	Logistic Regression	30.29 ± 20.16	71.70 ± 9.48	51.00 ± 12.17	53.10 ± 16.36
	SVM	25.55 ± 17.09	82.55 ± 11.74	54.05 ± 12.84	49.57 ± 14.76
	KNN	23.83 ± 21.59	66.00 ± 14.79	44.92 ± 9.23	41.89 ± 14.92
	Random Forest	30.84 ± 19.27	67.03 ± 10.78	48.94 ± 8.81	52.69 ± 12.57
	AdaBoost	41.00 ± 20.83	67.03 ± 9.30	54.02 ± 7.38	55.53 ± 11.58
	Gaussian NB	28.50 ± 17.89	87.16 ± 7.94	57.83 ± 11.04	51.37 ± 16.69
Whole Image + Background	Logistic Regression	79.81 ± 9.28	80.84 ± 10.30	80.32 ± 7.26	86.26 ± 7.21
	**SVM**	**85.51 ± 8.70**	**80.01 ± 8.54**	**82.76 ± 6.28**	**89.37 ± 4.64**
	KNN	68.27 ± 11.58	79.05 ± 11.12	73.66 ± 5.80	85.16 ± 5.14
	**Random Forest**	**73.63 ± 11.27**	**81.04 ± 14.16**	**77.34 ± 7.12**	**88.08 ± 6.64**
	AdaBoost	74.52 ± 10.77	82.83 ± 9.97	78.67 ± 4.01	84.19 ± 6.68
	Gaussian NB	68.20 ± 22.93	82.83 ± 10.78	75.52 ± 13.13	82.98 ± 10.21

Bold rows indicate the best performance.

## Data Availability

The original data presented in the study are openly available in MAMA-MIA at 10.1038/s41597-025-04707-4 (accessed on 23 October 2025).
